# Supplementary material for: Hospital Length of Stay and Surgery among European Children with Rare Structural Congenital Anomalies—A Population-Based Data Linkage Study
Source: Int J Environ Res Public Health. 2023 Mar 1;20(5):4387. doi: 10.3390/ijerph20054387 (PMC10002318; doi:10.3390/ijerph20054387)
Supplement: Supplementary file 1 [file ijerph-20-04387-s001.zip › ijerph-2177349-supplementary.pdf]

Supplementary Table S1: Number of children in the EUROlinkCAT study

	Number of children		Included in analysis of 0-4 years	Included in analysis of 0-9 years
	Reference children	EUROCAT children		
Finland	911,679	38,324	Yes	Yes
Denmark, Funen	100,748	2,423	Yes	Yes
UK, Wales	531,784	17,448	Yes	Yes
Italy, Tuscany	23,503	4,225	Yes	No
Italy, Emilia-Romagna	223,995	5,381	Yes	No
Spain, Valencian Region	168,563	4,260	Yes	No
UK, Thames Valley	Data for reference children not available	3,845	Yes	Yes
UK, Wessex		4,320	Yes	Yes
UK, East Midlands & South Yorkshire		11,278	Yes	Yes

Supplementary Table S2. Rules for surgery definitions

Include as surgery	Not include as surgery
Dilatations with or without stent implantation	Examinations/diagnostic procedure
Removal of foreign bodies from bronchus, lungs and oesophagus	Removal of foreign bodies in open areas (nose, ear, throat, skin)
Drainage from internal organs	Drainage with easy access by needle
Extraction of multiple teeth	Extraction of one tooth only, other dental treatment
Application of internal and external fixation to bone	Closed manipulation/application traction of bones
Harvest of skin, bone, tendon	Removal of suture, tube
	Attention, irrigation, aspiration
